# Improvement in peripheral airway function after a 12-week exercise intervention in COPD

**DOI:** 10.1186/s12931-026-03933-7

**Published:** 2026-10-12

**Authors:** Anna Cederberg, Hugo Öhrneman, Linda Elowsson, Ellen Tufvesson, Caroline Larsson, Bryan Falcones, Lisa Karlsson, Hamid Akbarshahi, Jaro Ankerst, Leif Bjermer, Andreas Palm, Jakob Löndahl, Andrei Malinovschi, Christer Janson, Margareta Emtner, Gunilla Westergren-Thorsson

**Affiliations:** 1 Lung Biology, Department of Experimental Medical Science, Faculty of Medicine, Lund University, Lund, Sweden; 2 Division of Ergonomics and Aerosol Technology, Department of Design Sciences, Lund University, Lund, Sweden; 3 Respiratory Medicine, Allergology, and Palliative Medicine, Department of Clinical Sciences, Faculty of Medicine, Lund University, Skåne University Hospital, Lund, Sweden; 4 Human Movement: Health and Rehabilitation, Department of Clinical Sciences, Faculty of Medicine, Lund University, Lund, Sweden; 5 Department of Medical Sciences: Respiratory, Allergy and Sleep Research, Uppsala University, Uppsala, Sweden; 6 Department of Medical Sciences: Clinical Physiology, Uppsala University, Uppsala, Sweden; 7 Department of Experimental Medical Science, Lund University, BMC C12, Lund, 221 84 Sweden

**Keywords:** Chronic obstructive pulmonary disease (COPD), Exercise therapy, Lung function, Impulse oscillometry, Airway mechanics

## Abstract

**Background:**

Exercise is a cornerstone of chronic obstructive pulmonary disease (COPD) management and improves quality of life, exercise capacity, exacerbation frequency and survival. However, the physiological mechanisms underlying these effects remain unclear. Small airway dysfunction and airway remodeling may not be adequately captured by spirometry, whereas impulse oscillometry (IOS) may be more sensitive to changes in respiratory mechanics. We hypothesized that an exercise intervention designed to engage the peripheral lung may promote adaptive, and potentially, reparative processes in COPD. The aim of the study was to evaluate changes in lung function after a 12-week exercise intervention, focusing on small airway-related measures assessed by IOS. Secondary aims were to assess changes in exercise capacity and patient-reported outcomes.

**Methods:**

In this prospective multicenter single-arm non-randomized intervention study, participants with COPD (*n* = 62) completed a 12-week supervised, individually tailored moderate-intensity aerobic- and muscle-strengthening exercise program (3 sessions/week). Lung function (spirometry, body plethysmography, diffusing capacity and IOS), exercise capacity (6-minut walk test, 1-minute sit-to-stand) and symptom burden (CAT, CCQ, mMRC) were assessed before and after the intervention.

**Results:**

Significant improvements were observed in IOS measures including R5 (median paired difference (Δ) = − 0.75 z-score, *p* = 0.001), R5–R20 (Δ=−0.18 z-score, *p* = 0.007), Fres (Δ=−0.33 Hz, *p* = 0.022) and AX (Δ=−0.05 kPa·s⁻¹, *p* = 0.042). No significant changes were observed in spirometry or lung volumes, except for a small increase in FVC (Δ = 0.12 z-score, *p* = 0.027). Exercise capacity improved (Δ6MWT = + 15 m, *p* = 0.002; Δ1STS = + 3 repetitions, *p* < 0.0001), while symptom burden decreased (ΔCAT = − 1.0, *p* = 0.014; ΔCCQ = − 0.1, *p* = 0.011).

**Conclusions:**

IOS measures improved after the exercise intervention, suggesting physiological adaptations beyond skeletal muscle conditioning that resulted in improved airway mechanics and ventilation distribution in the peripheral lung, while other lung function measures remained largely unchanged.

**Clinical trial registration:**

ClinicalTrials.gov ID NCT06335992 (Registration date 2024-03-28).

**Supplementary Information:**

The online version contains supplementary material available at https://doi.org/10.1186/s12931-026-03933-7.

## Background

Chronic obstructive pulmonary disease (COPD) is a growing global health problem and was ranked the fourth leading cause of death worldwide in 2024 [1]. Characterized by persistent airflow limitation due to airway obstruction and alveolar destruction, COPD causes symptoms such as dyspnoea, muscle wasting, and reduced physical capacity. These symptoms often lead to physical inactivity [2, 3]. Exercise is a cornerstone of long-term COPD management [4, 5] and has been shown to improve quality of life, exercise capacity, and clinical outcomes including reduced mortality, exacerbations and hospital admissions [5–7].

Despite these benefits, the physiological mechanisms underlying exercise induced effects on lung function remain poorly understood. Previous exercise intervention studies in COPD report little or no improvements in spirometric measures [8], which may partly reflect its limited sensitivity to pathophysiological changes in the peripheral lung. Airway narrowing, increased stiffness, inflammation and extracellular remodelling are key pathological features of COPD that contribute to peripheral lung abnormalities, including small-airway obstruction, loss of terminal bronchioles, alveolar destruction and emphysema. These changes lead to ventilation heterogeneity, gas trapping and increased physiological dead space [9]. While spirometry primarily reflects airflow limitation in the proximal airways, these peripheral abnormalities may be better captured using alternative measures such as diffusing capacity for carbon monoxide (DLCO) and respiratory oscillometry [10–12].

Impulse oscillometry (IOS) has emerged in recent years as a promising non-invasive method for detecting subtle changes in lung mechanics and identifying small airway dysfunction [13]. Low-grade systemic inflammation, including altered monocyte and lymphocyte profiles, has also been linked to impaired peripheral airway function assessed by IOS [14]. IOS measures impedance of the respiratory system by superimposing pressure oscillations at multiple frequencies during normal tidal breathing. Lower-frequency oscillations penetrate deeper into the lung and reflect resistance of both central and peripheral airways, whereas higher frequencies primarily assess central airway function. This frequency-dependent behaviour enables IOS to differentiate between proximal and distal airway contributions to overall respiratory mechanics [12, 15, 16].

Recent evidence further supports the sensitivity of oscillometric measures in detecting subtle changes in lung function and respiratory mechanics that may not be captured by spirometry [15, 16]. Despite this, relatively few exercise intervention studies in COPD have included lung function outcomes beyond spirometry, and the application of respiratory oscillometry in this context remains limited. We hypothesized that an exercise intervention designed to engage the peripheral lung may promote adaptive, and potentially, reparative processes in COPD, detectable as changes in respiratory mechanics. Therefore, evaluating changes in lung function using sensitive techniques such as IOS, alongside assessments of exercise capacity and patient-reported outcomes, may provide a more comprehensive understanding of the physiological adaptations to exercise in individuals with COPD.

The aim of this study was to investigate whether a 12-week exercise intervention is associated with functional and mechanical adaptations in the respiratory system in participants with COPD, with particular focus on peripheral airway mechanics assessed by IOS-derived measures of airway resistance, respiratory system reactance, and small airway dysfunction. Secondary aims were to assess changes in exercise capacity and patient-reported outcomes.

## Study design and methods

### Study design

**Fig. 1:**
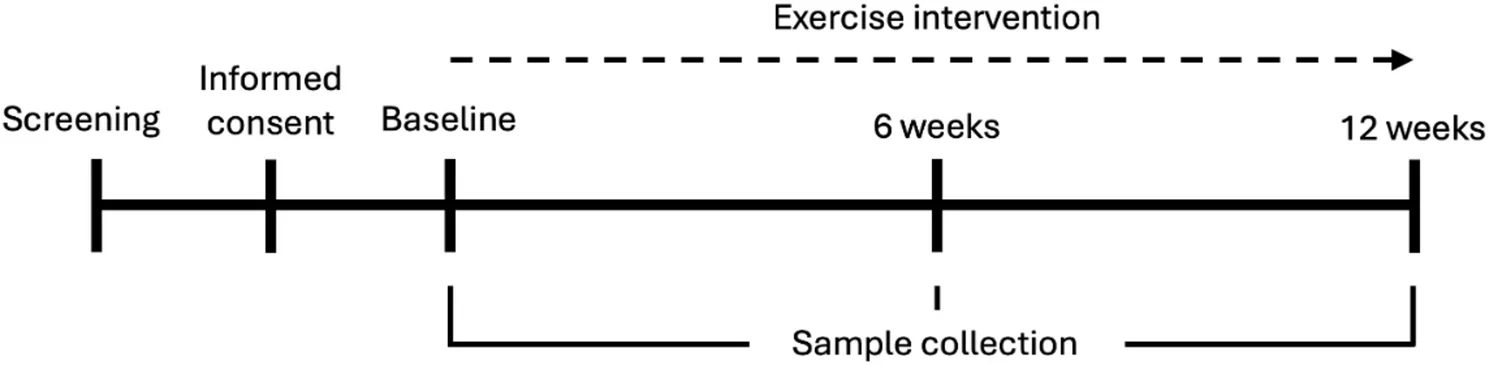
Schematic of trial design, intervention and sample collection

This prospective 12-week exercise intervention study reports baseline and longitudinal changes in lung function, exercise capacity, and patient reported outcomes. The design is shown in Fig. 1 and was described in detail previously [17]. The individual participant constituted both the unit of assignment and the unit of analysis. This study is reported in accordance with the TREND guidelines for Nonrandomized/Quasi-Experimental Study Designs [18]. The study was approved by the ethical review authority (dnr 2022-00831-01) and registered before recruitment began at ClinicalTrials.gov (ID NCT06335992, Registration date 2024-03-28).

### Participants and recruitment

Participants were consecutively recruited between 2022 and 2024 at two study sites in Lund and Uppsala, Sweden. Eligibility was determined through screening procedures and predefined inclusion/exclusion criteria, and the full eligibility criteria are available in the published study protocol [17]. Briefly, individuals with physician-diagnosed COPD, aged 50–85 years, ex-smokers (≥ 10 pack-years; smoking cessation ≥ 1 year), with post-bronchodilator FEV1 30–85% predicted and FEV1/FVC < 0.70, BMI 18–37 kg/m², without regular exercise participation (≥ 2 sessions/week) and an oxygen saturation of at least 87% during the 6-minute walk test (6MWT) were included. All participants provided written informed consent prior to inclusion.

Exclusion criteria included long-term oxygen therapy, ongoing oral corticosteroids/biologic treatment, recent exacerbations (within 6 weeks) or frequent exacerbations (> 1 in the previous 12 months), and unstable cardiovascular/pulmonary disease or other conditions preventing participation. Medical treatment followed the Global Initiative for Chronic Obstructive Lung Disease (GOLD) strategies [19] and the Swedish National Guidelines [20].

### Intervention description

A detailed description of the exercise intervention is found in the protocol paper [17]. The participants completed a 12-week intervention with three training sessions per week (twice supervised and one home-based). The programme was individually tailored to the participants’ symptoms, limitations and needs, with workload progressing over time as tolerated.

The supervised training on-site sessions consisted of interval aerobic training and resistance training. Interval aerobic training comprised 33-minute sessions of ergometer cycling at moderate intensity targeting > 60% W peak, and a perceived exertion of 4–6 on the Borg CR-10 scale. Resistance training comprised 30-minute sessions with 8–12 exercises targeting major upper and lower body muscle groups, performed as 3 sets of 8–12 repetitions. Home-based sessions included 30 min of self-chosen aerobic training (e.g. walking or cycling) and the same resistance exercises as performed on-site. Compliance was supported through the supervised sessions, individualized progression, and regular physiotherapist feedback.

### Study procedures

Study measurements were collected at baseline and after 6 and 12 weeks of exercise, as described in detail in the study protocol [17] and in Fig. 1. The primary outcome was change in IOS-derived peripheral airway function. Secondary outcomes included spirometry, exercise capacity, blood cell profile, and patient-reported outcomes.

### Assessments

#### Lung function tests

Spirometry, IOS, body plethysmography, fractional exhaled nitric oxide (FeNO), diffusing capacity of the lung for carbon monoxide (DLCO) and single-breath nitrogen wash-out were acquired before and after the exercise intervention. Additional measurements of spirometry and IOS were collected half-way during the exercise program (i.e. after 6 weeks).

Spirometry was performed post-bronchodilation according to American Thoracic Society and European Respiratory Society (ATS/ERS) guidelines [21] using a Jaeger MasterScope (Carefusion, Hoechberg, Germany). Forced expiratory volume in 1 s (FEV_1_), forced vital capacity (FVC), and the ratio between them (FEV_1_/FVC) were recorded.

IOS (Jaeger MasterScreen IOS, CareFusion, Hoechberg, Germany) was performed according to recommendations [13]. The resistance at 5 and 20 Hz (R5 and R20) were acquired, alongside reactance at 5 Hz (X5), the resonant frequency (Fres), and the area of reactance (AX).

Body plethysmography (MasterScreen Body, Erich Jaeger GmbH) was used to measure static lung volumes according ATS/ERS standards [22]. Total lung capacity (TLC), residual volume (RV), the ratio RV/TLC, functional residual capacity (FRC), inspiratory capacity, and total resistance (Rtot) were recorded.

*D*_LCO_ was measured using the MasterScreen Pulmonary Function Test (PFT) equipment (Erich Jaeger GmbH), according to ATS/ERS standards [23]. In addition, the alveolar volume (V_A_) and the carbon monoxide transfer coefficient (K_CO_) were recorded.

FeNO was measured using NIOX VERO^Ⓡ^(Areocrine, Sweden) or Ecomedics, CLD88 (Dürnten, Switzerland). The mean of three adequate measurements was recorded.

Single breath nitrogen wash-out was (N_2_MBW) performed using an Exhalyzer D (Eco Medics, Dürnten, Switzerland). The mean of the phase III slope (SIII) of three accurate measurements was recorded.

### Secondary outcome measures

#### Exercise capacity

The 6MWT and the 1-minute sit-to-stand test (1STS) were performed at baseline and post intervention according to established recommendations [24]. The best of two 6MWT was recorded to minimise learning effects. The minimal clinical important difference (MCID) is 30 m for the 6MWT [24, 25] and 3 repetitions for the 1STS [26].

#### Questionnaires

The questionnaire measurements were collected at baseline and after 6 and 12 weeks of intervention.

Symptom burden and health related quality of life were assessed using the COPD Assessment test (CAT) and the Clinical COPD questionnaire (CCQ) [27]. MCID is 2 points for CAT [28] and 0.4 points for CCQ [29].

Dyspnoea was assessed using the modified Medical Research Council (mMRC) scale, grading the dyspnoea from 0 to 4 [30].

### Sample size

The study was designed to include approximately 80 participants with clinically stable COPD, accounting for an anticipated dropout rate of 10–20%. Based on previous data [31], 15 participants were estimated to provide 80% power to detect improvements in endurance time during ergometer cycling.

### Data analysis and statistics

All lung function values are reported as absolute values, as percent of predicted values, or z-scores. Although predicted values from Hedenström [32, 33] were used for inclusion, those reference equations do not cover all lung function indices included in this study. Therefore, the Global Lung Initiative reference equations were used for result presentation of spirometry, body plethysmography, and diffusing capacity [34]. In two cases, the age of the participant exceeded the age range of the reference equations for body plethysmography. In these cases, the age was set to the maximal age (80 years) instead. For IOS, predicted values were acquired from the equations established by Oostveen et al. [35].

For statistical analysis, R (version 2024.04.2 + 764) and Python (version 3.10.7) were used. Analyses were based on participants who completed the intervention and available measurements. All tests used were non-parametric, including Wilcoxon’s singed rank test for comparisons and Spearman’s rank correlation for association between variables. All results are given as median and interquartile range. A P-value of 0.05 was considered statistically significant.

## Results

**Fig. 2:**
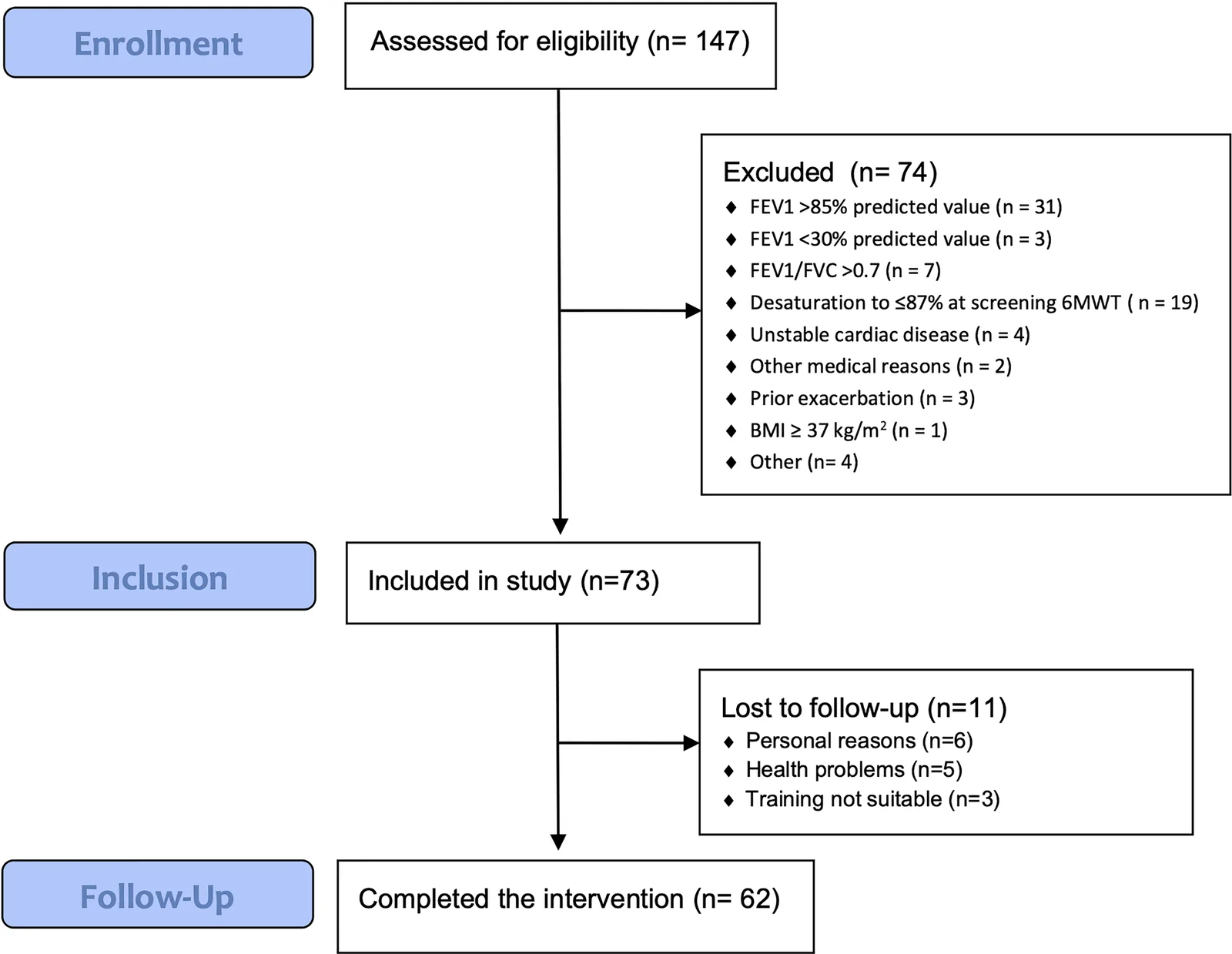
Flow chart of included participants

In total, 73 individuals were enrolled and 62 completed the intervention. The most common comorbidities were hypertension (13%) and diabetes (3%). Participant flow through the study is presented in Fig. 2 and the baseline characteristics of the cohort and demographic are described in Table 1. Five individuals dropped out before starting the intervention due to personal reasons, and six during the intervention due to health problems (*n* = 2), personal reasons (*n* = 1) and that the training did not suit them (*n* = 3). Median compliance with the 24 supervised aerobic sessions was 98.3% (range 83–100%).

**Table 1:** Characteristics of cohort, at baseline (pre-intervention) (*N* = 62)

	Pre-intervention
Age, median (IQR)	71.5 (68.0–76.0)
Sex, n males/females	27/35
BMI, median (IQR)	26.6 (25.0–30.5)
Pack years, median (IQR)	28.0 (19.2–39.5)
Comorbidities	
Hypertension, n (%)	8 (13)
Diabetes, n (%)	2 (3)
COPD Severity	
Mild (FEV_1_ ≥ 80% of predicted), n	16
Moderate (FEV_1_ 50–79% of predicted), n	37
Severe (FEV_1_ 30–49% of predicted), n	9
Very severe (FEV_1_ < 30% of predicted), n	0

### Lung function

**Fig. 3:**
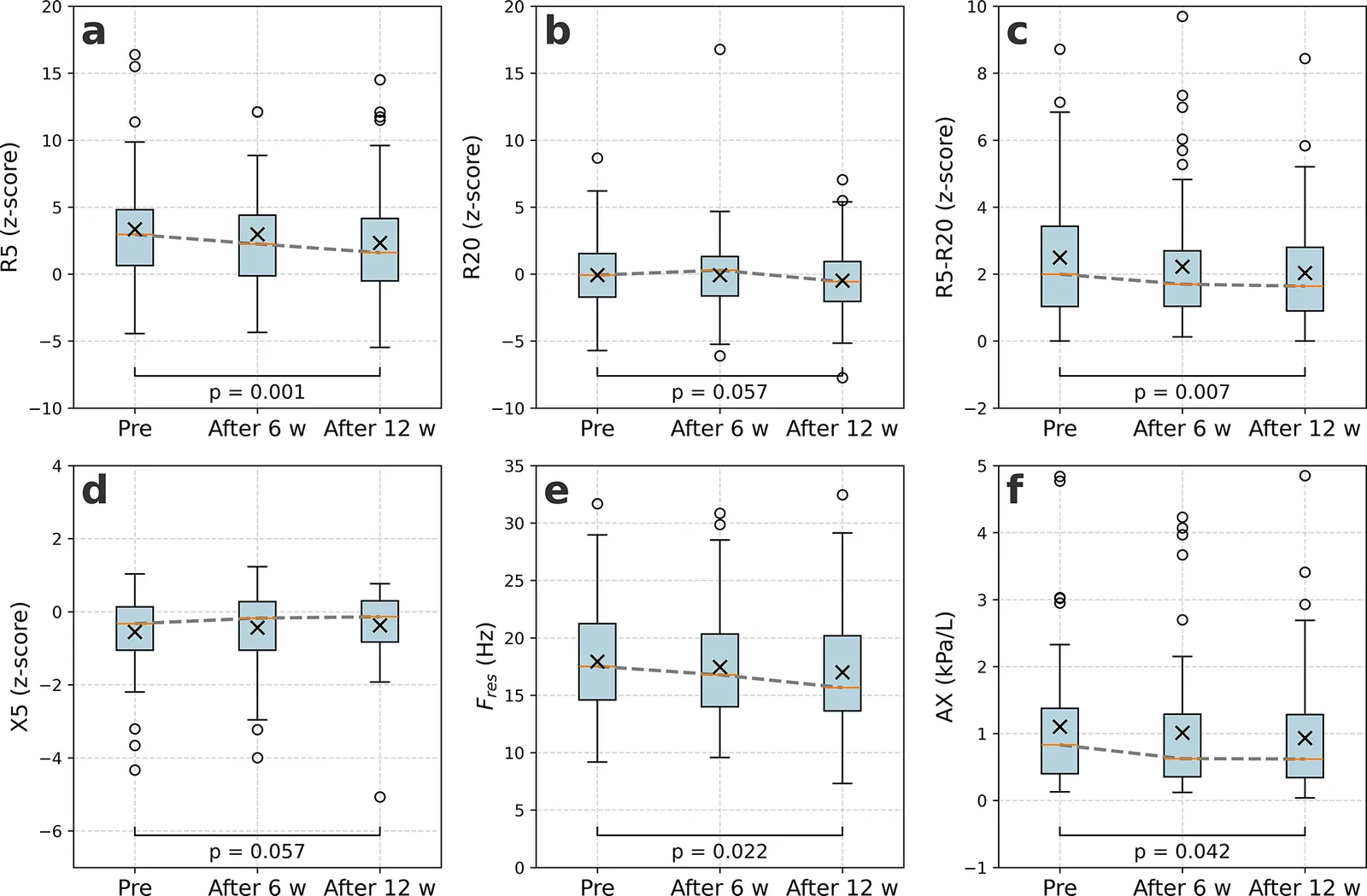
Impulse oscillometry results before, in the middle of, and at the end of the exercise program. Lines represent the median, crosses the mean and circles mark statistical outliers. Data is presented as z-scores for R5 (**a**), R20 (**b**), R5-R20 (**c**), and X5 (**d**), and as absolute values for Fres (**e**) and AX (**f**). R5: reactance at 5 Hz; R20: resistance at 20 Hz; X5: reactance at 5 Hz: Fres: resonance frequency: AX: area of reactance

IOS measurements showed a significant reduction in R5, R5-R20, Fres and AX after the 12-week exercise intervention. Most notably, the median R5 z-scores decreased from 2.96 at baseline to 2.24 and 1.60 after 6 and 12 weeks, respectively. In addition, the paired median differences were reduced for R5-R20 (Δ=−0.18, *p* = 0.007), Fres (Δ=−0.33 Hz, *p* = 0.022) and AX (Δ=−0.05 kPa·s⁻¹, *p* = 0.042). Median z-scores for R20 decreased and increased for X5 after the intervention (Figs. 2–3; Table 2, Table S1).

After the intervention, the paired median differences of both D_LCO_ and K_CO_ were significantly reduced by 0.20 and 0.15 standard deviations, respectively (Table 2). No significant changes were observed in N_2_ Single breath Washout or in FeNO (Table S1). FVC was marginally increased after the intervention, but no other significant changes in spirometry or lung volumes were observed (Table 2). All lung function measurements pre- and post-intervention are reported as absolute values in Table S1, and as % of predicted values in Table S2.

**Table 2:** Lung function measurements pre- and post-intervention, reported as z-scores. (*N* = 62)

	Pre-intervention	Post-intervention	Median paired difference	*P*-values
Spirometry				
FEV_1_ (z-score)	-1.58 (-2.31 – -1.20)	-1.54 (-2.15 – -1.06)	0.06 (-0.12–0.23)	0.063
FVC (z-score)	-0.34 (-0.83–0.23)	-0.17 (-0.75–0.25)	0.12 (-0.06–0.37)	0.027
FEV_1_/FVC (z-score)	-2.12 (-3.03 – -1.40)	-2.18 (-2.99 – -1.53)	-0.02 (-0.27–0.17)	0.280
Body Plethysmography				
TLC (z-score)	0.33 (-0.38–0.84)	0.23 (-0.40–0.98)	0.04 (-0.21–0.23)	0.958
VC (z-score)	-0.62 (-1.10–0.12)	-0.60 (-1.15–0.10)	-0.01 (-0.38–0.17)	0.347
RV (z-score)	0.66 (0.21–1.30)	0.70 (0.21–1.29)	-0.01 (-0.21–0.26)	0.930
FRC (z-score)	0.42 (-0.23–1.05)	0.51 (-0.16–1.29)	0.09 (-0.18–0.26)	0.270
IC (z-score)	-0.05 (-0.83–0.29)	-0.34 (-0.84–0.29)	0.01 (-0.39–0.26)	0.453
Impulse Oscillometry				
R5 (z-score)	2.96 (0.64–4.81)	1.60 (-0.50–4.16)	-0.75 (-2.63–0.38)	0.001
R20 (z-score)	-0.07 (-1.71–1.53)	-0.57 (-2.04–0.94)	-0.41 (-1.22–0.41)	0.057
R5-R20 (z-score)	2.00 (1.04–3.43)	1.64 (0.90–2.80)	-0.18 (-0.83–0.28)	0.007
X5 (z-score)	-0.32 (-1.05–0.13)	-0.14 (-0.83–0.30)	0.12 (-0.12–0.34)	0.057
Diffusing capacity of carbon monoxide				
D_LCO_ (z-score)	-1.72 (-2.40 – -1.04)	-1.81 (-2.90 – -1.00)	-0.20 (-0.36–0.10)	0.001
V_A_ (z-score)	-0.41 (-1.00–0.00)	-0.53 (-1.00 – -0.01)	-0.12 (-0.24–0.12)	0.232
K_CO_ (z-score)	-1.49 (-2.40 – -0.82)	-1.69 (-2.67 – -0.83)	-0.15 (-0.32–0.00)	< 0.001

FEV_1_: forced expiratory volume in 1 s; FVC: forced vital capacity; TLC: total lung capacity; VC: vital capacity; RV: residual volume; FRC: functional residual capacity; IC: inspiratory capacity; R5: reactance at 5 Hz; R20: resistance at 20 Hz; X5: reactance at 5 Hz; DLCO: Diffusing capacity of carbon monoxide; V_A_: alveolar volume; K_CO_: carbon monoxide transfer coefficient

### Blood cell profile

No significant changes were observed in blood differential cell count and haemoglobin values during the intervention (Table S3).

### Exercise capacity

After the intervention, a significant improvement in exercise capacity was detected in both the 6MWT and 1STS (Table 3). The median paired difference in walking distance was 15.0 (-5-37.8) m and 3.0 (0-4.3) repetitions of 1STS.

**Table 3:** Exercise tests pre- and post-intervention

	Pre-intervention	Post-intervention	Median paired difference	*p*-value
6MWD (m)	496 (361–638)	524 (376–679)	15 (-5.0–37.8)	0.002
1STS (repetitions)	21.0 (16.8–25.0)	22.5 (19.8–27.0)	3 (0.0–4.2)	< 0.0001

Results are given as median and inter-quartile range

6MWD: 6-minute walk distance (*N* = 60); 1STS: 1-minute sit-to-stand test (*N* = 56)

### Quality of life and symptom burden

**Fig. 4:**
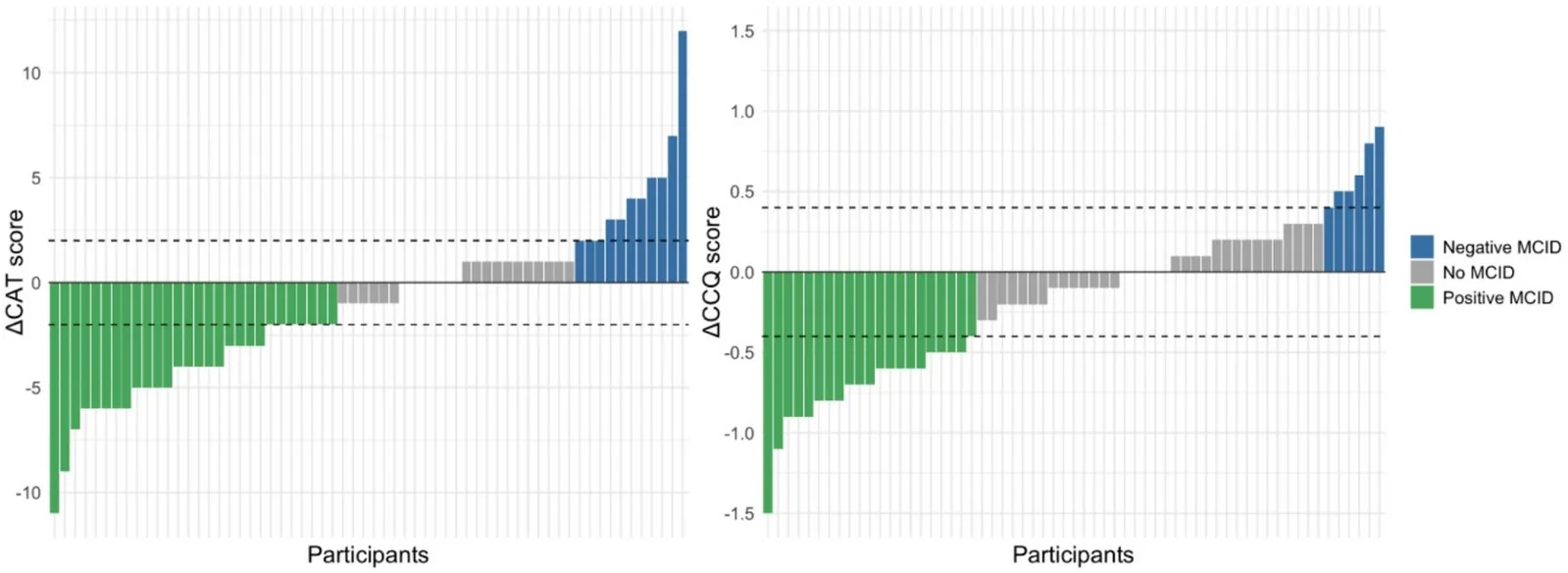
Individual differences in CAT and CCQ scores post-intervention compared to baseline. Each bar represents one participant (Green=Positive MCID, Grey = No MCID, Blue=Negative MCID), and the dashed line represents the MCID (±2 and ±0.4, respectively)

Improvements reaching MCID in patient-reported outcomes was observed in 28 of 62 of the participants for the CAT score and 20 of 61 in the CCQ score (Fig. 4) with a significant within-subject change (*p* = 0.014; *p* = 0.0111) (Table 4). Notably, participants with a high baseline score tended to have a greater reduction of perceived symptom burden, whereas those with lower baseline scores tended to report worsening for all three questionnaires (Figure S1).

**Table 4:** Symptom burden questionnaires pre- and post-intervention. (*n* = 62)

	Pre-intervention	Post-intervention	Median paired difference	*P*-value
CAT	12.0 (8.0–17.0)	12.0 (8.0–15.0)	-1.0 (-11.0 − 12.0)	0.014
CCQ	1.4 (0.9–1.8)	1.3 (0.8–1.6)	-0.1 (-1.50–0.90)	0.011
mMRC	1.0 (1.0–2.0)	1.0 (1.0–2.0)	0 (-2.00–2.00)	0.260

CAT: Chronic pulmonary obstructive disease assessment test; CCQ: Clinical COPD questionnaire (*n* = 61); mMRC: modified Medical Research Council scale

### Correlations

Apart from a marginal correlation between pair-wise changes in R5 and FEV1 (*r*=-0.26, *p* = 0.038), there were no clear associations between IOS results and other lung function or training outcomes. Significant correlations were however found between change in R5 and lymphocyte (ρ=-0.30, *p* = 0.020) and monocyte pair-wise change (ρ=-0.32, *p* = 0.012) (Table S4). No correlations were found between changes in lung function and changes in exercise capacity (Table S5).

## Discussion

In this study, we evaluated changes in lung function following a 12-week exercise intervention in participants with COPD, with a particular focus on the peripheral lung assessed by IOS. The intervention was associated with significant improvements in several IOS parameters reflecting airway resistance and reactance, indicating measurable changes in peripheral lung function. In contrast, only marginal changes were observed in spirometric measures. Additionally, exercise capacity improved, as demonstrated by increases in both the 6MWD and 1STS tests, accompanied by a significant within-subject reduction in symptom burden based on questionnaires. In line with previous studies [8], the exercise intervention produced only marginal changes in spirometry. In contrast, clear and consistent improvements were observed in several IOS parameters, most prominently in reductions of R5 and R5-R20. These changes suggest reduced respiratory resistance and less frequency-dependent resistance, indicating less uneven ventilation and improved peripheral airway mechanics. This interpretation is further supported by the significant reductions in Fres and AX, indicating improved respiratory system reactance and reduced peripheral airway dysfunction. X5 also tended to increase, a pattern compatible with improved elastic properties and reduced peripheral airway closure. In parallel, R20 showed a tendency to decrease, which may suggest that the observed mechanical improvements were not restricted to the peripheral airways but also have involved larger conducting airways. These findings are consistent with our hypothesis that exercise may promote functional and mechanical adaptations in the peripheral lung.

Evidence from healthy volunteers shows that low‑intensity aerobic exercise can induce immediate reductions in airway resistance and improvements in IOS parameters [10], while studies in COPD demonstrate that IOS is sensitive to detect dynamic changes in airway resistance and reactance during exercise in response to bronchodilation [36]. In our study, the observed reductions in airway resistance and improved reactance therefore suggest improved mechanical properties of the peripheral airways, which may represent early indicators of improved small-airway function. By increasing ventilatory demand and promoting deeper breathing at higher lung volumes [37], targeted exercise may impose mechanical forces on the airways and lung parenchyma, providing a plausible mechanism by which exercise could influence airway mechanics and tissue adaptation. Such mechanical forces may contribute to reopening of unstable small airways, reduced ventilation heterogeneity, and improved lung compliance. At the cellular level, mechanical stimulation can be sensed by structural cells and may influence cellular signaling, extracellular matrix organization and tissue stiffness, processes relevant to airway remodeling and repair [38]. Overall, the IOS findings are consistent with physiological benefits of exercise and suggest broader improvements in airway mechanics after exercise that were not detected by spirometry alone. Although these changes may be compatible with adaptive processes in the peripheral airways, the underlying mechanisms remain to be clarified in future studies incorporating molecular and structural assessments.

In contrast to improvement in the IOS data, a small decrease in both KCO and DLCO was observed after the intervention. Although these reductions were modest, decreasing by at most 0.2 standard deviations on the group-level, they were unexpected. As V_A_ is unchanged, these decreases are unlikely to be the result of altered ventilation distribution, despite an apparent improvement in FVC. While these results are difficult to explain, the small absolute effect within the reproducibility bias of the DLCO method makes the results unlikely to be clinically relevant.

Associations were found between changes in R5 and circulating monocyte and lymphocyte counts, suggesting that improvements in respiratory resistance may have coincided with subtle systemic immune adaptations. This interpretation is supported by recent findings showing that physical activity in COPD is associated with immune-regulatory changes in circulating lymphocytes [39]. Unlike previous studies, FEV_1_ did not predict the improvement in exercise capacity, which demonstrate associations between lung function and 6MWT performance [40–42]. However, FEV₁ typically correlates poorly with patient‑reported symptoms and functional limitation in COPD [43, 44]. This underscores the multidimensional nature of COPD and supports the view that rehabilitation outcomes are influenced by factors extending beyond lung function, and especially spirometry, alone. Given the large number of correlations examined, there is a risk of false positives (type I errors), and therefore statistically significant associations should be interpreted cautiously.

We aimed to have a well-defined study cohort by applying strict inclusion and exclusion criteria, with particular focus on recruiting participants who did not engage in regular exercise and who had limited comorbidities. As most participants had a mild to moderate COPD, with few classified as severe COPD, baseline lung function was relatively high. This inherently reduced the likelihood of detecting large improvements in lung function, but at the same time increased confidence that any observed changes were directly related to the intervention. Although individuals with more severe COPD may respond differently to exercise-based intervention, no clear indications of this were observed in this study. In addition to a relatively homogenous group, the study population was rather small. Furthermore, the findings can only be applied to the specific type and duration of the intervention, which limits the generalisability of the results. Another limitation ensuring full adherence to the training programme during unsupervised home-based sessions. Even during supervised sessions, variation in individual effort is inevitable and may have contributed to variability in the observed outcomes.

## Conclusion

In conclusion, the present study showed that a 12-week exercise intervention in individuals with COPD was associated with improved peripheral airway function as assessed by IOS, increased exercise capacity and reduced symptom burden. While conventional spirometric measurements remained largely unchanged, the improvements across several IOS parameters highlight the sensitivity of this technique for detecting functional changes in the small airways that may otherwise be overlooked. These findings support the concept that exercise induces physiological benefits beyond skeletal muscle conditioning, with changes indicative of improved airway mechanics and ventilation distribution. The absence of a clear relationship between baseline lung function and training response underscores the heterogeneity of COPD and suggests that exercise-based interventions may be beneficial across the range of disease severity represented in this cohort.

## Supplementary Information

Below is the link to the electronic supplementary material.

Supplementary Material 1

## Data Availability

Involved researchers will have access to anonymized study data. Access for external parts to the datasets used and/or analysed during the current study will available be available in collaboration with responsible study investigator on reasonable request.

## Abbreviations

ATS/ERS: American Thoracic Society and European Respiratory Society
COPD: Chronic obstructive pulmonary disease
FEV1: Forced expiratory volume in one second
FVC: Forced vital capacity
IOS: Impulse oscillometry
1STS: 1-minute sit-to-stand test
6MWT: 6-minute walk test
