# Supplementary material for: Improvement in peripheral airway function after a 12-week exercise intervention in COPD
Source: Respir Res. 2026 Oct 12;27:394. doi: 10.1186/s12931-026-03933-7 (PMC13656524; doi:10.1186/s12931-026-03933-7)
Supplement: Supplementary file 1 [file 12931_2026_3933_MOESM1_ESM.docx]

**Online- only Supplemental Material**

**Table S1**: Lung function measurements pre- and post-intervention, reported as absolute values. Results are given as median and inter-quartile range. p-values were calculated using Wilcoxon’s signed rank test.

| *Spirometry* | **Pre-intervention** | **Post-intervention** | ***P*-values** |
| --- | --- | --- | --- |
| FEV_1_ (L) | 1.84 (1.49–2.06) | 1.80 (1.49–2.18) | 0.066 |
| FVC (L) | 3.10 (2.74–3.71) | 3.23 (2.77–3.83) | **0.014** |
| FEV_1_/FVC | 0.70 (0.54–60.14) | 0.69 (0.53–61.70) | 0.456 |
| *Body Plethysmography* |  |  |  |
| Rtot (kPa s L^-1^) | 0.28 (0.23–0.37) | 0.29 (0.21–0.36) | 0.394 |
| TLC (L) | 6.05 (5.27–6.90) | 5.96 (5.34–6.82) | 0.953 |
| VC (L) | 3.37 (2.77–3.85) | 3.31 (2.80–3.80) | 0.350 |
| RV (L) | 2.71 (2.31–3.09) | 2.68 (2.28–3.15) | 0.965 |
| RV/TLC (%) | 43.9 (35.7-52.2) | 43.11 (33.4-52.9) | 0.577 |
| FRC (L) | 3.66 (3.13–4.02) | 3.63 (3.16–4.18) | 0.199 |
| IC (L) | 2.45 (2.06–2.89) | 2.37 (2.11–2.75) | 0.459 |
| *Impulse Oscillometry* |  |  |  |
| R5 (kPa s L^-1^) | 0.37 (0.30–0.48) | 0.34 (0.29–0.44) | **0.001** |
| R20 (kPa s L^-1^) | 0.28 (0.24–0.33) | 0.28 (0.23–0.32) | **0.036** |
| R5-R20 (kPa s L^-1^) | 0.09 (0.06–0.14) | 0.07 (0.04–0.12) | **0.008** |
| X5 (kPa s L^-1^) | -0.15 (-0.22–-0.11) | -0.15 (-0.19–-0.10) | 0.051 |
| F_Res_ (Hz) | 17.52 (14.59–21.23) | 15.66 (13.64–20.19) | **0.022** |
| AX (kPa s^-1^) | 0.83 (0.40–1.38) | 0.62 (0.34–1.28) | **0.042** |
| *Diffusing capacity of carbon monoxide* |  |  |  |
| D_LCO_ (mmol min^-1^ kPa^-1^) | 5.33 (4.22–6.30) | 5.10 (3.97–6.14) | **0.002** |
| V_A_ (L) | 5.01 (4.23–5.68) | 4.88 (4.27–5.62) | 0.234 |
| K_CO_ (mmol min^-1^ kPa^-1^ L^-1^) | 1.04 (0.91–1.20) | 1.00 (0.85–1.19) | **< 0.001** |
| *N_2_ Single breath Washout and Fractional Exhaled Nitric Oxide* | | | |
| Phase III slope (L^-1^) | 5.3 (3.8-7.9) | 4.7 (3.6-7.9) | 0.425 |
| Fractional Exhaled Nitric Oxide (ppb) | 14.7 (10.6-24.4) | 17.5 (11.3-25.5) | 0.928 |

n = 62 for all values with exception of N_2_ Single breath Washout (n = 58) and Fractional Exhaled Nitric Oxide (n = 55). FEV_1_: forced expiratory volume in 1 s; FVC: forced vital capacity; TLC: total lung capacity; VC: vital capacity; RV: residual volume; FRC: functional residual capacity; IC: inspiratory capacity; R5: reactance at 5 Hz; R20: resistance at 20 Hz; X5: reactance at 5 Hz; FRes: resonance frequency; AX: area of reactance; D_LCO_: Diffusing capacity of carbon monoxide; V_A_: alveolar volume; K_CO_: transfer coefficient for carbon monoxide (D_LCO_/V_A_).

**Table S2**: Lung function measurements reported as percentage of the predicted value, pre- and post-intervention. Results are given as median and inter-quartile range. p-values were calculated using Wilcoxon’s signed rank test.

|  | **Pre-intervention** | **Post-intervention** | ***P*-value** |
| --- | --- | --- | --- |
| *Spirometry* | |  |  |
| FEV_1_ (% of predicted) | 73.1 (61.0–80.0) | 72.9 (62.0–82.8) | 0.064 |
| FVC (% of predicted) | 94.3 (87.1–104.0) | 97.1 (87.9–104.1) | 0.023 |
| FEV_1_/FVC (% of predicted) | 76.9 (65.1–85.9) | 77.1 (63.8–84.3) | 0.282 |
| *Body Plethysmography* |  |  |  |
| TLC (% of predicted) | 104.1 (95.2–111.8) | 103.1 (94.7–113.7) | 0.908 |
| VC (% of predicted) | 92.2 (85.8–101.5) | 92.3 (85.5–101.4) | 0.364 |
| RV (% of predicted) | 118.8 (106.3–140.6) | 120.9 (106.0–139.0) | 1.000 |
| RV/TLC (% of predicted) | 116.1 (106.8–127.8) | 116.6 (106.4–130.5) | 0.651 |
| FRC (% of predicted) | 108.9 (95.5–123.1) | 110.9 (96.7–128.0) | 0.218 |
| IC (% of predicted) | 99.0 (83.6–105.8) | 93.8 (82.9–105.9) | 0.444 |
| *Impulse Oscillometry* |  |  |  |
| R5 (% of predicted) | 126.6 (106.5-155.7) | 114.5 (95.9-144.9) | 0.001 |
| R20 (% of predicted) | 99.2 (84.2-113.3) | 96.0 (82.3-107.9) | 0.042 |
| R5-R20 (% of predicted) | 247.3 (-137.3-934.6) | 243.2 (-137.3-756.9) | 0.204 |
| X5 (% of predicted) | 121.7 (89.8-164.0) | 110.1 (83.1-155.7) | 0.062 |
| *Diffusing capacity of carbon monoxide* | | | |
| D_LCO_ (% of predicted) | 74.0 (62.6–83.6) | 71.7 (58.4–83.4) | 0.001 |
| V_A_ (% of predicted) | 95.0 (87.7–100.0) | 93.5 (87.5–99.8) | 0.251 |
| K_CO_ (% of predicted) | 76.9 (65.3–88.0) | 75.1 (60.7–87.2) | < 0.001 |
| V_A_ / TLC | 0.84 (0.76–0.88) | 0.84 (0.76–0.88) | 0.864 |
| FEV_1_: forced expiratory volume in 1 s; FVC: forced vital capacity; TLC: total lung capacity; VC: vital capacity; RV: residual volume; FRC: functional residual capacity; IC: inspiratory capacity; R5: reactance at 5 Hz; R20: resistance at 20 Hz; X5: reactance at 5 Hz; D_LCO_: Diffusing capacity of carbon monoxide; V_A_: alveolar volume; K_CO_: transfer coefficient for carbon monoxide (D_LCO_/V_A_). | | | |

**Table S3:** Hemoglobin and blood cell counts values pre- and post-intervention. Results are given as median and inter-quartile range. p-values were calculated using Wilcoxon’s signed rank test.

|  | **Pre-intervention** | **Post-intervention** | ***P*-value** |
| --- | --- | --- | --- |
| Haemoglobin (g/L) | 142.0 (133.3–148.8) | 139.5 (134.0–146.0) | 0.0768 |
| Leukocytes (×10⁹/L) | 7.00 (5.33–8.38) | 6.90 (5.43–7.90) | 0.3791 |
| Neutrophiles ( ×10⁹/L) | 4.50 (3.00–5.20) | 4.20 (2.92–5.07) | 0.4192 |
| Eosinophiles (×10⁹/L) | 0.11 (0.08–0.20) | 0.11 (0.08–0.20) | 0.4232 |
| Basophiles (×10⁹/L) | 0.01 (0.00–0.04) | 0.00 (0.00–0.03) | 0.3865 |
| Lymphocytes (×10⁹/L) | 1.70 (1.50–2.00) | 1.60 (1.40–2.08) | 0.9083 |
| Monocytes (×10⁹/L) | 0.60 (0.40–0.70) | 0.55 (0.40–0.70) | 0.8811 |

**Table S4:** Spearman correlation between the percentage change in R5 and the percentage change in other lung function and blood cell counts variables. Correlations with p-values above 0.05 have been omitted.

|  | **Spearmans rho** | ***P*-value** |
| --- | --- | --- |
| Monocytes | -0.3172 | 0.012 |
| Lymphocytes | -0.2952 | 0.020 |
| Lymphocytes | -0.2724 | 0.032 |
| Rtot | 0.2677 | 0.035 |
| FEV_1_(%pp) | -0.2641 | 0.038 |
| FEV_1_ | -0.2595 | 0.042 |
| Rtot (%pp) | 0.2516 | 0.049 |
| FRC: functional residual capacity; FEV_1_: forced expiratory volume in 1 s; TLC: total lung capacity; Rtot: total airway resistance. | | |

**Figure S1.** Baseline questionnaire scores correlated to the change in questionnaire scores. CAT score (r=-0.29; p =0.023), CCQ score (r=-0.56; p < 0.0001), mMRC score (r=-0.48, p < 0.0001). n = 62 (CAT, mMRC), n =61 (CCQ). CAT: Chronic pulmonary obstructive disease assessment test; CCQ: Clinical COPD questionnaire; mMRC: modified Medical Research Council scale.

**Table S5:** Spearman correlation between the *change* in all variables and the change in 6MWT, results with p-values above 0.05 have been omitted.

|  | **Spearmans rho** | ***p*-value** |
| --- | --- | --- |
| R_in_ (% predicted) | 0.320 | 0.013 |
| SIII (from N_2_MBW) | 0.312 | 0.015 |
| SIII % pred (from N_2_MBW) | 0.289 | 0.025 |
| CAT score | -0.290 | 0.024 |
| 6MWT -pulse after test | 0.261 | 0.044 |
| R_in_: Inspiratory resistance; SIII (from N_2_MBW): phase III slope from nitrogen multiple-breath washout; CAT: Chronic pulmonary obstructive disease assessment test; 6MWT: 6-minute walk test. | | |
